# The Effect of Surface Modification of Ti13Zr13Nb Alloy on Adhesion of Antibiotic and Nanosilver-Loaded Bone Cement Coatings Dedicated for Application as Spacers

**DOI:** 10.3390/ma12182964

**Published:** 2019-09-12

**Authors:** Magda Dziaduszewska, Marcin Wekwejt, Michał Bartmański, Anna Pałubicka, Grzegorz Gajowiec, Tomasz Seramak, Anna M. Osyczka, Andrzej Zieliński

**Affiliations:** 1 Biomaterials Division, Department of Materials Engineering and Bonding, Gdańsk University of Technology, 80-233 Gdańsk, Poland; 2 Department of Laboratory Diagnostics and Microbiology with Blood Bank, Specialist Hospital in Kościerzyna, 83-400 Kościerzyna, Poland; 3 Department of Surgical Oncology, Medicial University of Gdańsk, 80-210 Gdańsk, Poland; 4 Department of Biology and Cell Imaging, Institute of Zoology and Biomedical Research, Faculty of Biology, Jagiellonian University, 30-387 Kraków, Poland

**Keywords:** spacer, coating adhesion, Ti13Zr13Nb alloy, selective laser melting, bone cements modification

## Abstract

Spacers, in terms of instruments used in revision surgery for the local treatment of postoperative infection, are usually made of metal rod covered by antibiotic-loaded bone cement. One of the main limitations of this temporary implant is the debonding effect of metal–bone cement interface, leading to aseptic loosening. Material selection, as well as surface treatment, should be evaluated in order to minimize the risk of fraction and improve the implant-cement fixation the appropriate manufacturing. In this study, Ti13Zr13Nb alloys that were prepared by Selective Laser Melting and surface treated were coated with bone cement loaded with either gentamicin or nanosilver, and the effects of such alloy modifications were investigated. The SLM-made specimens of Ti13Zr13Nb were surface treated by sandblasting, etching, or grounding. For each treatment, Scanning Electron Microscope (SEM), contact profilometer, optical tensiometer, and nano-test technique carried out microstructure characterization and surface analysis. The three types of bone cement i.e., pure, containing gentamicin and doped with nanosilver were applied to alloy surfaces and assessed for cement cohesion and its adhesion to the surface by nanoscratch test and pull-off. Next, the inhibition of bacterial growth and cytocompatibility of specimens were investigated by the Bauer-Kirby test and MTS assay respectively. The results of each test were compared to the two control groups, consisting of commercially available Ti13Zr13Nb and untreated SLM-made specimens. The highest adhesion bone cement to the titanium alloy was obtained for specimens with high nanohardness and roughness. However, no explicit relation of adhesion strength with wettability and surface energy of alloy was observed. Sandblasting or etching were the best alloys treatments in terms of the adhesion of either pure or modified bone cements. Antibacterial additives for bone cement affected its properties. Gentamicin and nanosilver allowed for adequate anti-bacterial protection while maintaining the overall biocompatibility of obtained spacers. However, they had different effects on the cement’s adhesive capacity or its own cohesion. Furthermore, the addition of silver nanoparticles improved the nanomechanical properties of bone cements. Surface treatment and method of fabrication of titanium affected surface parameters that had a significant impact on cement-titanium fixation.

## 1. Introduction

Spacers are instruments used in revision surgery for local control of postoperative infection. Usually, this is a temporary implant made of antibiotic-loaded bone cement or it consists of a metal rod covered with bone cement containing an antibiotic [1]. These procedures have recently become the gold standard for the treatment of bone infection with a high, near 90% effectiveness [2,3]. The need of using spacers results from the lack of systematic antibiotherapy efficacy and the necessity to ensure mobility of the patient [4]. Spacers can bought commercially bought or manually prepared by applying bone cement containing antibiotic on a metal rod, sometimes using a special mold. The following main types of spacers can be distinguished: revision hip or knee endoprosthesis spacers or intramedullary nail spacers [1,2,3,4,5]. Literature reports that the overall complication rate of using spacers is about 26%. The most common complications after spacer implantation include the dislocation of a spacer, fracture of implant or surrounding bones, erosion in bones, and systemic antibiotic toxicity. Their main risk factors are: loss of bone support as a results of improper spacer design, incorrectly prepared cements and overall patient’s state of health and functioning [5,6]. Moreover, one of the primary problems with using spacers is related to the debonding effect of the metal–bone cement interface, where micro-sliding movements between the components lead to aseptic loosening [7,8]. The prevention of aseptic loosening requires the appropriate manufacturing, material selection, as well as surface treatment of spacers.

In numerous studies [9,10,11] it has been suggested that implant-cement fixation properties might be improved by surface roughness augmentation of the metal rod, which exposes sharp-edges texture, enlarges effective surface, and chemically activates it. The ideal metal and cement joining interface should rely on the association of micromechanical and chemical bonding [12]. A wide variety of rod surface modification techniques have been developed regarding the adhesion strength, including: blasting, acid-etching, grinding, oxidizing, plasma-spraying, as well as coating application [13,14]. Mostly, blasting, acid-etching, and grinding are used, due to their convenient and widespread commercial implication [15,16,17]. Moreover, it is assumed that the bactericidal modifications of bone cement can also affect the adhesion of the cement. Antibiotics, such as gentamicin or ciprofloxacin, are widely used, however they affect the mechanical properties of bone cement [18]. Hence, nanosilver recently is an intensively tested alternative. Its main advantage is high bactericidal effectiveness, resistance to polymerization temperature, as well as no adverse effect on the mechanical properties of the cement. 

For the longevity of spacers, the appropriate material selection is another important issue. The use of a metal rod can significantly increase the strength and stability of the spacer [1,4]. So far, the primary metal used for that kind of medical solution was corrosion-resistant steel. However, due to low biocompatibility and high density negatively affecting its weight, titanium alloys have commonly substituted corrosion-resistant steel. Among others, titanium with zirconium and niobium deserves special attention, due to their excellent biocompatibility, corrosion resistance, high strength/weight ratio, good fatigue resistance, and lower Young’s modulus as compared to most of the metals, thus better matching the values of those existing in human bones [17,19]. 

Last but not least, the ability to create personalized spacer might result in their longevity. Hence, selective laser melting (SLM), which shows several advantages over conventional manufacturing techniques, including highly accurate and predictable structure, customized mechanical properties, personal-customized architectures, might be an interesting option [20]. Giving the importance of surface topography, the SLM process seems to have the prominent ability to obtain satisfied parameters of roughness. However, it may also produce unmelted particles after processing [21]. Those particles might have a negative effect on mechanical properties of the rod, or eventually they may loosen, which can have a significant impact on metal–bone cement interface. For this reason, post-processing, such as sandblasting or chemical etching, is usually implemented [22]. Although some authors provide the evaluation of adhesion strength of two dental adhesives in the laboratory under shear or tensile modes [23], the problem of adhesion strength between the metal/bone cement still seems to be poorly explored. Other studies evaluate shear adhesive strength [16], or tensile strength of different materials, mostly metal/ceramic [24]. To our knowledge, there are no reports on the adhesive joining between SLM-made specimens and cement.

The main aim of this research was to investigate the effect of surface modifications of Ti13Zr13Nb alloy on the adhesion of pure and modified bone cement. We evaluated the titanium surface after different surface treatment, assessed cement adhesion to this surface, and checked the biological properties of modified bone cements. A commercially available titanium solid rod and SLM-produced were used as controls. Subsequently, the printed specimens were subjected to the following surface treatments: sandblasting, grinding, and etching. Three types of bone cement were applied to the prepared titanium substrates, i.e., pure, containing gentamicin and doped with nanosilver. Designing the proper shape of the titanium rod obtained by SLM method, application of surface treatment, and selection of an effective bactericidal bone cement coating were the key approaches for eliminating potential complications during implantation as well as ensuring the success of two-stage revision treatment. 

## 2. Materials and Methods

### 2.1. Specimens Preparation

#### 2.1.1. Titanium Specimens Fabrication

**Figure 1:**
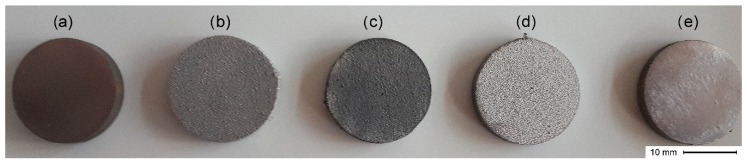
Images of the alloy specimens after different surface treatments (**a**) Solid bar; (**b**) Untreated selective laser melting (SLM); (**c**) Sandblasted SLM; (**d**) Etched SLM; and, (**e**) Ground SLM.

The cylindrical specimens (discs) were manufactured by SLM machine while using Ti13Zr13Nb spherical powder (TLS Technik GmbH & Co. Spezialpulver KG, Bitterfeld-Wolfen, Germany) with particle size ranging from 20 to 70 µm. Two sets of alloy samples were designed while using Materialise Magics (Materialise NV, Ghent, Belgium) software in the form of specimens suitable for surface evaluation (d = 20 mm, h = 3 mm) and biological tests (d = 10 mm, h = 3 mm). The cylinders were oriented in the following combination: the smaller ones in parallel position, while the bigger ones perpendicularly to the building platform. The alloy specimens were manufactured by the Realizer SLM 100 (Realizer GmbH, Borchen, Germany) machine that was equipped with the ytterbium one mode fiber laser CW YLR-100-SM (IPG Laser GmbH, Burbach, Germany) using 1070 nm wavelength. The process parameters (i.e., spot diameter, laser power and scanning speed, and layer thickness) are patent pending. Additionally, the laser melting process was carried under a protective argon atmosphere (grade 4.8). As a reference material, a hot-rolled bar of Ti13Zr13Nb alloy provided by a commercial supplier was used (Xi’an SAITE Metal Materials Development Co., Ltd., Xi’an, China). The rod specimens (d = 20 mm, h = 3 mm) were divided into five groups according to the surface treatment that was applied (Figure 1). The first group, which was made of the commercial solid bar (Solid bar), was used as a control A, while the second group were surface untreated SLM-made specimens (Untreated SLM), designated as a control B. Group 3 consisted of the SLM-made specimens that were treated by sandblasting (Sandblasted SLM) with aluminum oxide particles (50 µm) for 20 s and a distance of 10 mm from the sandblaster nozzle tip (d = 2 mm) with the working pressure 6 bars. Group 4 consisted of discs acid-etched for 15 min. in solution of nitric acid (65%) and hydrofluoric acid (40%) at ratio 3:1 (Etched SLM). The discs were then rinsed in distilled water for 10 min. in an ultrasonic bath and left to dry for 24 h. Group 5 consisted of ground SLM-made cylinders (Ground SLM). Manual grinding was based on the wet process with the use of the 220 µm (P80) grade silicon carbide paper that was mounted on a lapping machine Saphir 330 (ATM GmbH, Mammelzen, Germany) at a feeding rate of 400 rpm. Each sample was ground until the SLM porous structure was removed. Specimens of each group were finally cleaned in 95% ethanol while using an ultrasonic cleaner for 10 min. and left to dry in room temperature for 24 h.

#### 2.1.2. Cement Coating Preparation

The commercially available PMMA bone cement (Tecres, Verona, Italy) was used to create the coating on the titanium alloy specimens. Cement was prepared following the manufacturer’s instructions and according to the international standard ISO 5833:2002 [25]. The procedure consisted of manually combining the liquid component with the powder in a bowl and then mixing to a paste with a spatula for about one minute. After mixing, the paste was applied on titanium alloy specimens that were placed in specially prepared silicone moulds. Afterwards, it was allowed to cure for 1 h in ambient conditions. Three different cements were investigated: (1) unmodified bone cement (BC), (2) antibiotic-loaded bone cement (gentamicin sulfate, Sigma Aldrich, Steinheim, Germany) (A-BC), and (3) bone cement modified with nanosilver (MkNano, Mississauga, ON, Canada—average particles size 50 nm, purity 99.9%) (NpAg-BC). For the modified bone cement, the procedure included the addition of modifiers to the cement powder and then mixing for one min. The proposed modifications and their concentrations were selected based on the previous studies [26,27,28]. Table 1 presents the final compositions of the tested bone cements.

### 2.2. Surface Evaluation of Titanium Specimens

#### 2.2.1. Surface Topography

The surface topography of titanium alloy specimens after surface treatments was observed using a high-resolution Scanning Electron Microscope SEM (JEOL JSM-7800F, Tokyo, Japan). Moreover, the X-ray Energy Dispersive Spectrometer (EDS) investigated its chemical composition (Edax Inc., Mahwah, NJ, USA)

#### 2.2.2. Surface Roughness

The surface roughness of the titanium alloy specimens was assessed by using a contact profilometer with EVOVIS software (Hommel Etamic Waveline, Jenoptik, Jena, Germany). The test was conducted using cut-off wavelength λc =2.5 µm, evaluation length ln = 12.5 mm and scanning speed of 0.50 mm/s, according to ISO 4287-1997 [29]. The average values of roughness (Ra), the peak-to-valley roughness (Rz) and maximum peak-to-mean height (Rp) were obtained from this test. 

#### 2.2.3. Surface Wettability and Surface Energy

The surface wettability and surface energy (Ɣs) were determined by the contact angle measurements. The studies were carried out by the falling drop method with two liquids: distilled water and diiodomethane (Sigma Aldrich, Steinheim, Germany) while using an optical tensiometer (Attention Theta Life, Biolin Scientific, Espoo, Finland). The volume of the liquids was about 1 μL/sample. Each measurement was carried out five times, immediately after deposition of the drop of the liquid. The analysis was carried out while using the OneAttension program (Biolin Scientific, Espoo, Finland) and mathematical calculations that are based on the OWRK method [30,31,32].

#### 2.2.4. Surface Nanomechanical Properties

Nanomechanical properties of titanium alloy specimens were determined while using nanoindentation technique performed with the NanoTest™ Vantage (Micro Materials, Wrexham, UK) equipment. Three-sided diamond, pyramidal Berkovich indenter was used. Twenty-five (5 × 5) independent nanoindentation measurements on each of tested surface were performed. The maximum force od indentation was 50 mN, the loading and unloading time were set up to 20 s and holding time with maximum force was 5 s. The distance between the indentations was 20 μm for both axes (x and y). During a single measurement, the load-displacement curve was determined based on the Oliver and Pharr method [33]. The surface hardness (H) and reduced Young’s modulus (Er) were calculated while using the integrated software.

### 2.3. Assessment of Cement Cohesion and its Adhesion to the Surface of Titanium Specimens

#### 2.3.1. Cohesion of Cement

The cohesion of cements was determined while using nanoscratch-test performed with the NanoTest™ Vantage (Micro Materials, Wrexham, UK) equipment. The Berkovich indenter was used for nanomechanical properties investigation. Ten measurements of tested bone cement coating were performed. The applied load range of a single test was from zero to 200 mN with force rate 1.3 mN/s at a distance of 500 µm. During the measurements, the applied force in functional of friction force curve was determined. Based on the abrupt change in frictional force, the cohesion of tested cements was determined. 

#### 2.3.2. Adhesion of Cements to the Titanium Surface

**Figure 2:**
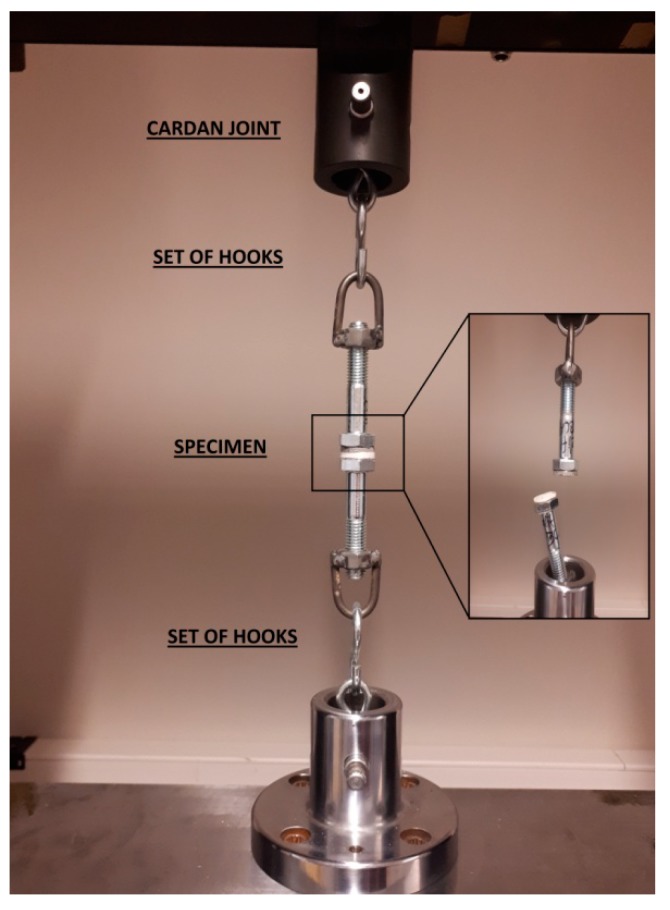
The specimen holder mounted in universal tensile machine.

The adhesive strength of bone cement coatings to the surface-modified titanium alloy specimens was tested according to the pull-off test procedure that was proposed by Ozturk & Tannant [34]. The purpose of the test was to determine the interface bond strength i.e., between cement coating and titanium specimens. For this reason, the greatest tensile stress that the surface area can bear before being detached was investigated. The bone cement coating with a thickness of 2 mm was applied on the titanium specimens in disc form (d = 20 mm, h = 3 mm). Next, the specimens were glued with a two component epoxy resin Metacryl (Technicqll, Trzebinia, Poland) to the flat threaded screws (M12) set in a linear manner. The screws and specimen had both been ground and cleaned while using acetone. After the epoxy was cured (24 h), the specimens were installed in the universal testing machine INSTRON 1195 (INSTRON, Norwood, MA, USA). The special holders contained cardan joint and a set of hooks was used in order to minimize the bending moments (Figure 2). The measurements were carried out with load at a constant rate of 1 mm/min. The maximum force was recorded upon failure and assumed as the adhesive strength, while the failed surface was visually examined and documented. 

### 2.4. Assessment of Biological Properties of Bone Cement Coatings

#### 2.4.1. Inhibition of Bacterial Growth

The inhibition of bacterial growth by bone cement coatings was evaluated by test of Bauer-Kirby et al. [35]. Two bacterial reference strains were used: *S. aureus* (ATCC 29213, Microbiologics Inc., St. Cloud, MN, USA) and *E. coli* (ATCC 25922, Microbiologics Inc., St. Cloud, MN, USA). Each bacteria strain was incubated in a sterile 0.9% NaCl solution for 10 min. Subsequently, 100 μL bacteria suspensions containing 1.5 × 10^8^ CFU mL^−1^ were taken and seeded on Mueller-Hinton Agar plates (Becton Dickinson GmbH, Heidelberg, Germany). The inhibition zone tests involved the incubation at 36 °C of the titanium specimens that were covered with bone cement coatings with the resulting bacterial suspension. The experiment was performed while using three specimens (n = 3) for each type of bone cement coating applied on titanium specimens (Untreated SLM) in disk form (d = 10 mm, h = 3 mm). Before testing, the bone cement specimens were sterilized in 70% EtOH, followed by 30 min. exposure to UV light. The measurements of the inhibition zone were carried out after: 24, 72, and 168 h. The bacterial growth inhibition zone was determined as a diameter without bacterial growth in mm. 

#### 2.4.2. Cytocompatibility

All of the reagents that were used in this study were purchased from Thermofisher (Thermofisher Scientific, Waltham, MA, USA), unless stated otherwise. The MTS assay was used in order to assess the cytocompatibility of tested bone cement coatings. For the assay, the mouse osteoblast precursor cell line MC3T3-E1 (ATCC CRL-2593, Manassas, VA, USA) was used. The cells were expanded in culture while using alpha-MEM supplemented with 15% fetal bovine serum (FBS), 0.1 mM l-ascorbic acid phosphate (Sigma Aldrich, Steinheim, Germany), and 1% antibiotics (penicilin-streptomycin—10,000 U/mL, Thermo Fisher Scientific, Waltham, MA, USA). When the cells reached a confluent monolayer, they were lifted from culture flasks while using 0.25% Trypsin-EDTA, counted and seeded directly on the surface of the cements, as described below. Briefly, three specimens for each type of bone cement (n = 3) in disk form (d = 20 mm, h = 2 mm) were used. Before testing, the bone cement specimens were sterilized in 70% EtOH followed by 30 min. exposure to UV light. Afterwards, the specimens were placed in separate wells of 24-well culture plates and were covered by 2 × 10^4^ MC3T3-E1 cells suspended in 1 mL of standard culture medium. The cells were incubated at 37 °C in air containing 5% CO_2_. The medium was aspirated and replaced with a fresh one every 48 h. Cell viability was evaluated while using CellTiter96 Aqueous One Solution Cell Proliferation Assay (MTS, Promega, Kraków, Poland). After three and seven days of culture, culture media were withdrawn and each material sample was covered with 400 μL of MTS solution diluted 10× in phenol-red free alpha-MEM. The plates were then incubated at 37 °C in a culture incubator until the development of a brownish color of the MTS solution. The intensity of the developed color is proportional to the number of actively metabolizing live cells. Approximately after 30 min., 200 μL aliquots of the MTS solutions from individual wells were transferred to separate wells in 96-well plates and their absorbance at 492 nm was colorimetrically measured while using a plate reader. The results were expressed as % change in MTS absorbance values for modified bone cements compared to 100% value assumed for pure bone cement. 

### 2.5. Statistical Method

Statistical analysis of the data was performed while using commercial software (SigmaPlot 14.0, Systat Software, San Jose, CA, USA). The Shapiro-Wilk test was used to assess the normal distribution of the data. All of the results were presented as mean ± standard deviation (SD) and they were statistically analyzed while using one-way analysis of variance (one-way ANOVA). Multiple comparisons versus the control group between means were performed while using the Bonferroni t-test with the statistical significance set at *p* < 0.05. Two controls of Ti13Zr13Nb were adopted in the statistical analysis: (A) specimens from commercially purchased bar (Solid bar) and (B) specimens that were produced by SLM (Untreated SLM).

## 3. Results and Discussion

### 3.1. Surface Evaluation of Titanium Alloy Specimens

#### 3.1.1. Surface Topography Evaluation

**Figure 3:**
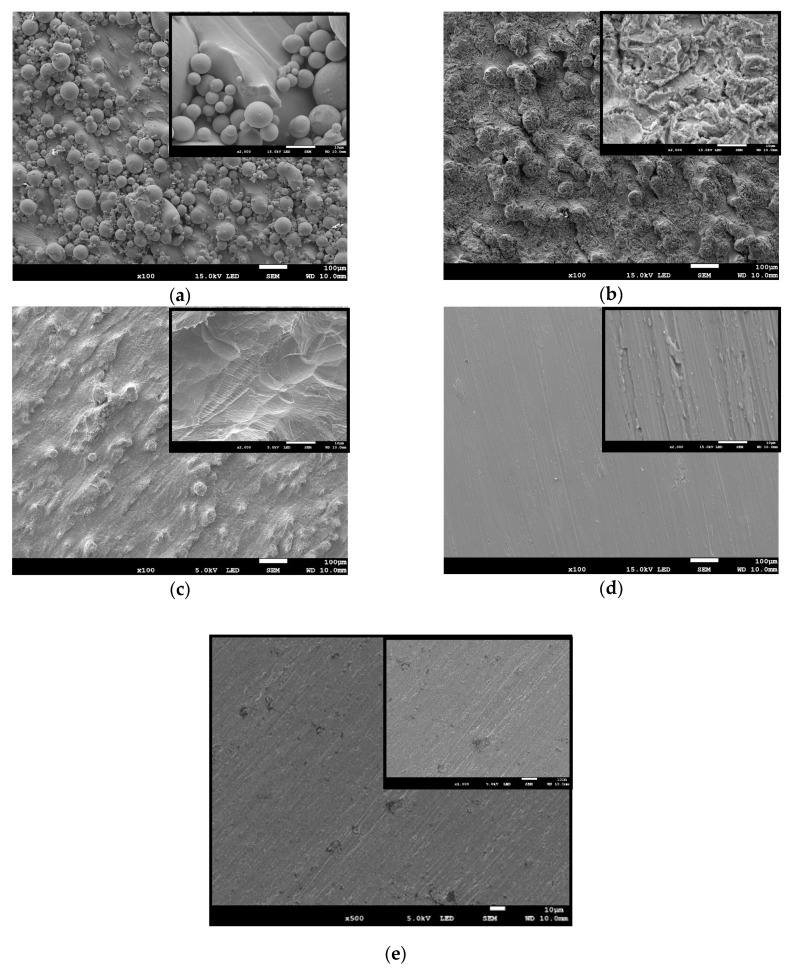
SEM images of the titanium alloy specimen surfaces after different surface treatments and at two different magnifications (×500 and ×2000): (**a**) Untreated SLM; (**b**) Sandblasted SLM; (**c**) Etched SLM; (**d**) Ground SLM; and, (**e**) Solid bar (the presented pictures are representative for five specimens).

**Figure 4:**
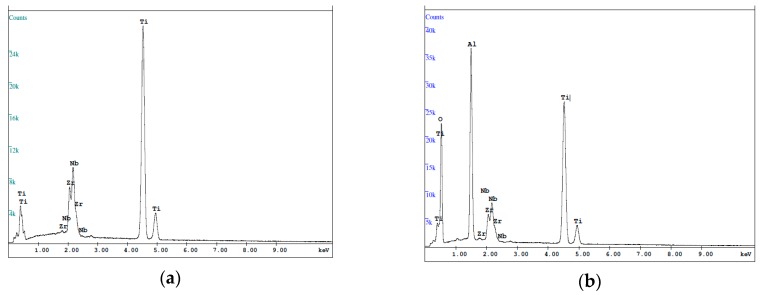
Results of Energy Dispersive Spectrometer (EDS) analysis after different surface treatments: (**a**) Solid bar, Untreated SLM, Etched SLM, Ground SLM; and, (**b**) Sandblasted SLM (the presented pictures are representative for three analyses of each surface treatment specimens).

In the control group, a commercial bar appeared to have the smoothest surface compared with other groups. Small pits and shallow grooves generated in the grinding procedures could be observed (Figure 3e). In opposite, SLM-made specimens exhibited the surface covered by numerous unmelted spherical particles of initial powder of various sizes. Most of the bigger grains consisted of the solid linchpin on the surface, while the smaller were grouped or loosely covered the specimen’s surface or other non-melted particles (Figure 3a). In the case of sandblasting treatment, the stream of accelerated particles removed most of the spherical particles and significantly expanded the surface with many irregular micro-cracks (Figure 3b). However, it could be noticed that some bigger particles were still mounted to the ground. Moreover, the EDS analysis of Sandblasted SLM specimens revealed the presence of alumina, likely from the abrasives after the sandblasting process (Figure 4). The etching treatments resulted in the formation of a more uniform fine-textured titanium alloy surface with most of the spherical grains being dissolved. This surface was relatively smoother and irregularities with well define depression areas were more evenly distributed (Figure 3c). The ground SLM specimens had relatively flat surface with clearly-visible shallow grooves generated by grinding and characteristic for small grade carborundum paper. However, the morphology revealed protruding elevations and depressions that were composed of several layers characteristic for SLM-made specimens (Figure 3d). As often reported by others, the SLM process causes irregularity due to internal porosity. For example, Fousova M. et al. [36] observed the 0.78 ± 0.11% internal porosity of fully dense samples. Such porosity of studied samples might be a result of processing technology, and/or different sizes of titanium alloy powder. 

#### 3.1.2. Surface Roughness Evaluation

A comparison of the mean roughness parameters: the average roughness (Ra), the peak-to-valley roughness (Rz) and maximum peak-to-mean height (Rp) values among the five experimental groups of titanium specimens that were obtained by surface profilometry testing are given in Table 2.

The lowest mean surface roughness value was obtained for control Solid bar (0.14 μm) and Ground SLM (1.38 μm), and it was significantly lower than for all other specimens tested. Statistically significant (*p* < 0.05) higher roughness was detected for all types of SLM-made specimens: without surface treatment (10.13 μm), sandblasted (10.79 μm), etched (8.82 μm), and ground (1.38 μm). Sandblasted and etched modifications had significantly increased the Ra parameter when compared to control specimens (Solid bar and Untreated SLM). However, the highest value of Ra was observed for sandblasting, while etching decreased the mean roughness parameters as compared to non-modified SLM specimens. Furthermore, the peak-to-valley roughness and maximum peak-to-mean height were in agreement with Ra values, merely the Rz and Rp of Ground SLM are not statistically significant when compared to Solid bar. 

These obtained results are consistent with SEM images (Figure 3), in which better surface development was observed due to various modifications. In the case of unmodified SLM, roughness was mainly created by unmelted powder particles. The obtained results are in line with the literature, where the values of Ra for SLM-made specimens are between 5 μm ≤ Ra ≤ 40 μm [37,38,39] and they mainly depend on the SLM processing and the properties of the powder [38]. Increasing values of roughness parameters for the specimens that were subjected to sandblasting are associated with the existence of the additional craters, ridges, and sharp edges of alumina particles [40]. Those plastic deformations accounted, among others, for the highest values of Rp (30.52 μm) [41]. Similarly, Aparicio C. et al. [42] reported that the sandblasted specimens were found to possess a rougher surface (4.74 μm) when compared to those after etching (1.69 μm). This might be explained by the action of the acid that removes most of the blasted material. However, Ra values were lower than those found by us, but this could be attributed to the various particles size, the shape or hardness of the blasting material [40]. Overall, it is assumed that the surface roughness increases with increasing ceramic particles size [43]. The surface roughness can be divided into three levels: macro-roughness (Ra ~ 10 µm), micro-roughness (Ra ~ 1 µm), and nano-roughness (Ra < 200 nm) [44,45]. Thus, it may be concluded that sandblasting and etching results in macro-roughness of specimens, whereas grinding results in specimen micro-roughness.

#### 3.1.3. Surface Wettability and Surface Energy Evaluation

Table 3 illustrates the surface wettability results of investigated modifications of titanium alloy specimens. 

The titanium alloy specimens that were produced by SLM were characterized by hydrophobic surface (120.3° > 90°). Applied surface modifications improved the surface wettability and resulted in the hydrophilic properties. The commercial titanium rod also had a hydrophilic surface (76.4). Overall, it is assumed that materials better adhere to hydrophilic surfaces [46]. Moreover, lower surface energies of adhesives should allow for their better adhesion and good bonding [47]. Giving a constant surface energy of cements, their adhesion will depend on the surface energy of titanium alloy specimens. Table 4 shows the surface energy of investigated specimens.

Based on the surface energy analysis, Etched SLM should be the best for adhesion and the Ground SLM should be the worst. For the most specimens, the adhesion will be mainly based on dispersive interactions. The only exception is Ground SLM, where dispersive and polar interactions occurred reasonably equally. 

#### 3.1.4. Surface Nanomechanical Properties Evaluation

**Figure 5:**
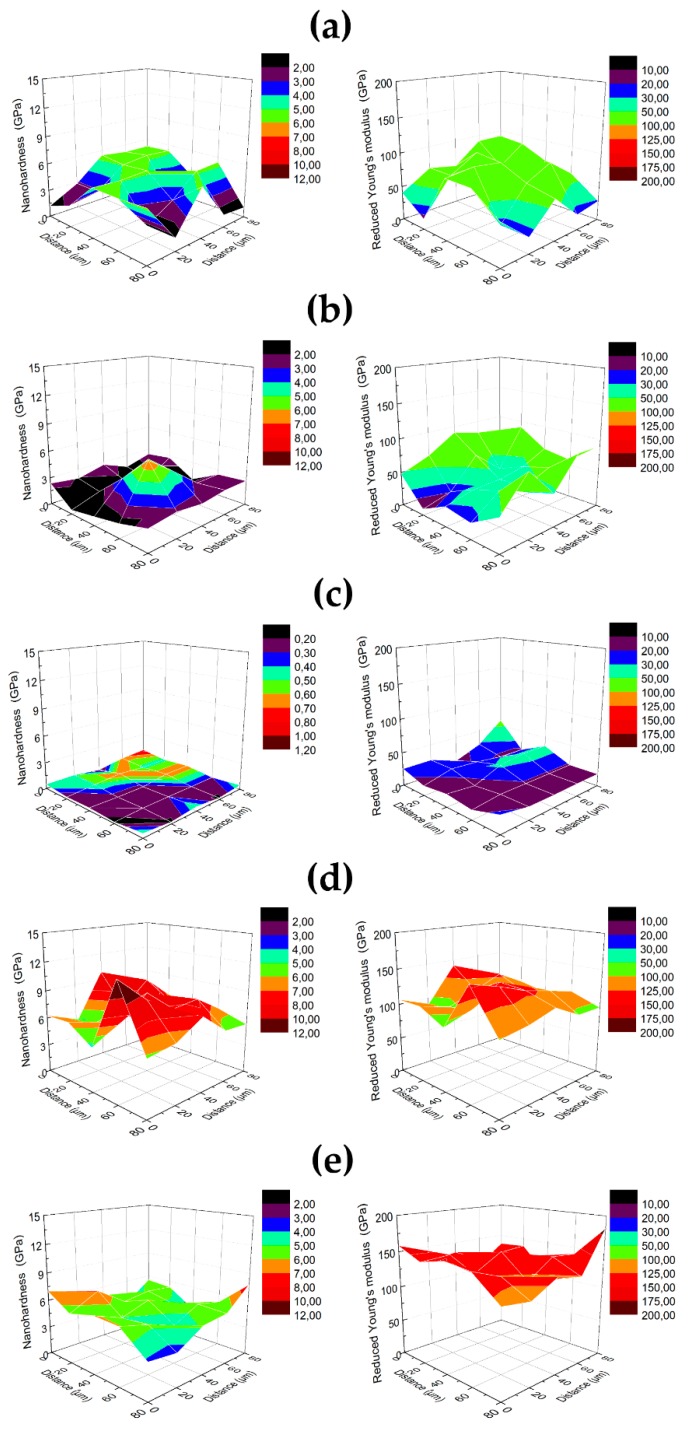
3D maps of nanomechanical (nanohardness—left and Reduced Young’s modulus—right) properties of studied specimens after different surface treatments: (**a**) Untreated SLM; (**b**) Sandblasted SLM; (**c**) Etched SLM; (**d**) Ground SLM; and, (**e**) Solid bar.

Table 5 shows the results of nanomechanical tests (nanohardness, Reduced Young’s modulus H/E and H^3^/E^2^ ratio). The nanohardness and Reduced Young’s modulus were shown in Figure 5 as three-dimensional (3D) maps. The highest nanomechanical values were observed for control specimens (6.77 GPa and 5.15 GPa for Untreated SLM and Solid bar, respectively). This may be due to stresses in the surface layer of these specimens that occur in the material after the grinding process. The appearance of the stress in a layer of material after grinding was proven by Tao et al. [48]. The smallest values of nanohardness and Reduced Young’s modulus were obtained for Etched SLM (0.41 GPa). The etching involves concentrated acids that change the surface topography and deteriorate the mechanical properties of specimens. Along with the increase in surface roughness values, the increasing standard deviation values are observed, which confirms the reliability of the tests that were carried out [49]. Rough surfaces are characterized by a non-uniform distribution of mechanical properties. Differences in roughness values and their impact on the standard deviation values of nanomechanical measurements correlate well with the graphic presentation of the results in Figure 5. The control surface (Solid bar) with the lowest roughness parameter Ra (0.14 µm) is characterized by the smallest relative standard deviation (~20% of value) and the most homogeneous distribution of results that are presented in Figure 5e. According to nanoindentation test, Etched SLM is the most suitable for implanting to the human body, because of the value of Young’s modulus (24 GPa) closest to that of a human bone (10–30 GPa) [50,51]. It is highly recommended that Young’s modulus of implants should be similar to the human bone [52]. Low mechanical properties of a surface layer might have a negative impact on the implantation process, because such a layer might be damaged during the implantation procedure. The nanoindentation method is also used to determine the resistance to failure of tested surface, which is defined by H/Er ratio [53]. A higher ratio reflects better resistance to elastic stress [54]. In addition, the H^3^/Er^2^ ratio indicates resistance to plastic deformation [55]. The results in the Table 5 clearly show that the Untreated SLM and Ground SLM specimens display the best wear resistance (H/E 0.066 and 0.060, H^3^/E^2^ 0.027 GPa and 0.020 GPa, respectively). The smallest value of the H/Er and H^3^/Er^2^ ratio were obtained for Etched SLM (0.018 and 0.0002 GPa, respectively), which was also characterized by the lowest values of the nanomechanical properties.

Table 6 presents the cohesion of bone cement coatings. The antibiotics did not significantly increase cohesion of the coatings (from 82.1 GPa to 84.9 GPa) opposite to the addition of nanosilver (from 81.2 mN for BC cement to 122 mN for NpAg-BC). Nanosilver doping into cements (NpAg-BC) had a significant and positive effect on their nanomechanical properties. This may be due to strengthening effect of metallic nanoparticles or the creation of a composite cement matrix. The improvement of bone cement mechanical properties by silver nanoparticles was previously reported by us [27]. Russo et al. also obtained similar effects [56] for gold nanoparticles. However, the presence of metallic nanoparticles in bone cements could deteriorate the mechanical properties of such composites due to stress concentration at the matrix/filler interface [57,58]. Besides, silver nanoparticles are probably able to form permanent bonds with matrix that improve the resistance of cements.

### 3.2. Creation of Bone Cement Coating on Titanium Specimens

Pure and modified cements were both successfully applied to all tested titanium alloy specimens. We noticed that the type of titanium alloy affects the cement adhesion. The most problematic in terms of their application were solid bar specimens (control A). Furthermore, there was no difference in the process of application of the coating for pure cement and doped with modifications. 

### 3.3. Assessment of Cement Adhesion to the Surface of Titanium Specimens

The results of our study indicate the phenomena that the increase of the surface roughness increases the effective area that is available for the better adhesion of cement. The SLM-made specimens: untreated, sandblasted and etched demonstrated significantly higher adhesion of either BC, A-BC, or NpAg-BC cements than solid titanium (control A). Similar results were obtained for the ground group covered by BC and NpAg-BC. Only SLM ground specimen with pure bone cement (BC) showed no significant difference when compared to the control A group (*p* < 0.05). All of the studied cement types displayed significantly lower adhesion to solid and SLM ground specimens as compared to untreated SLM specimens (control B), while sandblasting and etching of titanium alloys significantly increased the adhesion of BC (*p* < 0.05) and the modifications of BC by antibiotic or nanosilver did not affect this process. The addition of gentamicin and silver nanoparticles to BC also resulted in their better adhesion to untreated SLM, but they adhered poorer to the ground titanium alloys when compared to the non-modified BC. Furthermore, cement with antibiotic increased the pull-off-strength adhesion for solid titanium specimens, while the addition of nanosilver decreased. It would be ideal if the failure only appeared at the cement-titanium interface, but a very strong adhesion between BC and sandblasted and etched titanium alloys displayed higher than glued fixation. Overall, the debonding force for studied specimens was high enough to treat the titanium/cement adhesion as sufficient (Table 7).

The high standard deviation of the results can be explained by the methodology of the applied epoxy test, which is highly randomized and it depends on surface roughening, cleaning, drying, fixation time and glue mixing speed, epoxy-ingredients proportions, time of glue drying, position of drying, etc. 

**Figure 6:**
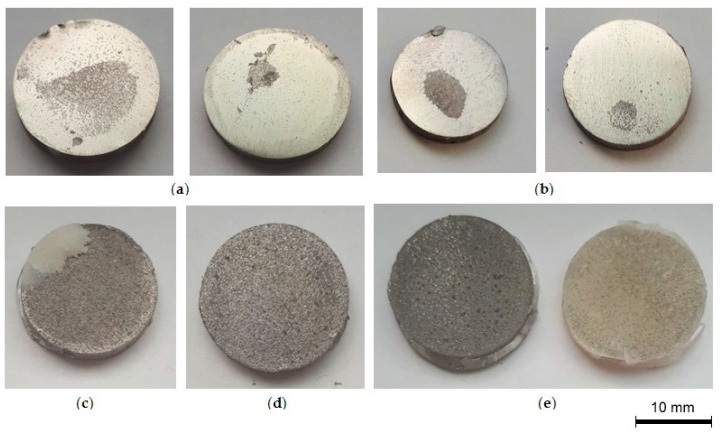
Images of selected specimens after detachment of bone cement coatings by pull-off-test (**a**) Solid bar; (**b**) Ground SLM; (**c**) Untreated SLM; (**d**) Etched SLM; and, (**e**) Sandblasted SLM (the presented pictures are representative for five experiments).

The highest adhesion of BCs to untreated SLM, as well as sandblasted and etched specimens, is associated with their roughness. Topographical and chemical properties of the coatings both might have contributed to the pull-off-strength results. Appropriate roughness enlarges the actual interface between the metal surface and cement, and thus increases the adhesive strength [59,60]. The alumina particles remaining after sandblasting provide mechanical interlocking, which improves the bonding strength with BC [61]. The oxide layer that formed after the etching of titanium alloys also improved the adhesion to BC. The slightly lower value of BC adhesion for untreated SLM might result from unmated titanium particles loosely covering the surface and thus decreasing the titanium/cement bonding. Weak adhesion between the bone cements and ground titanium alloys correlated with the lack of mechanical interlocking and their low roughness. Some authors [62,63,64] showed the correlation between coating adhesion to SLM-made specimens as compared to solid material. Different types of fracture images (adhesive, cohesive, and mixed) were observed in the samples after the detachment of the coating by pull-off-test (Figure 6).

The solid and ground specimens showed some detachment of bone cement. The cement delamination occurred, followed by the failure of the coating adhesion to the substrate. This suggested uneven adhesion of the cements to the metal. Cement islands of various size, numbers, and shape characterized the modified titanium alloy surfaces. After the pull-off test, the titanium alloy surfaces were enricher mostly with BC islands, less A-BC, and the smallest amounts NpAg-BC was found on titanium alloy surfaces. This confirms the results of the pull-off strength test, where the adhesive strength is lower. There were also morphological differences in fracture mode between untreated, sandblasted and etched SLM specimens as compared to the ground and solid ones. The untreated, sandblasted, and etched SLM disks were regularly covered by cement, not in the form of cement island, and this could be correlated with higher roughness and better adhesion strength of these titanium alloy specimens (Figure 6).

### 3.4. Assessment of Biological Properties of Bone Cement Coating

#### 3.4.1. Inhibition of Bacterial Growth

**Figure 7:**
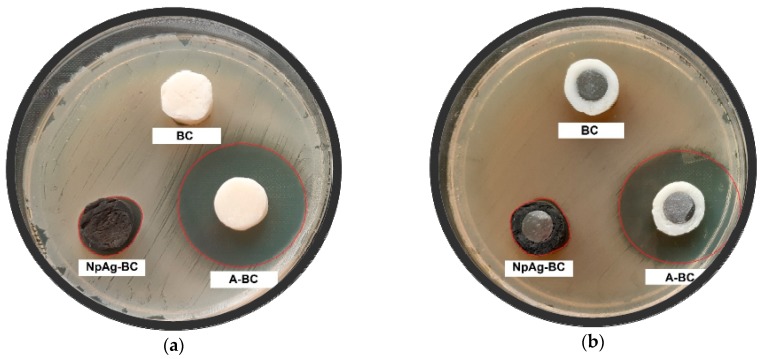
Bacterial growth inhibition on untreated SLM titanium alloy specimens coated with bone cements: unmodified (BC), antibiotic-loaded (A-BC) and modified with nanosilver (NpAg-BC) after 7 days of bacteria culture (the presented pictures are representative for three experiments): (**a**) *Staphylococcus aureus* and (**b**) *Escherichia coli.*

The occurrence of the inhibition zones indicated that the modified cements effectively inhibited bacterial growth (Figure 7).

Pure cement had no antibacterial properties. The addition of gentamicin or silver nanoparticles to BC inhibited local bacterial growth (Figure 7, Table 8).

There bacteria growth inhibition was substantially higher (about 10 mm zone) for A-BC than NpAg-BC (about 1 mm). Both of the modifiers effectively inhibited bacterial growth for seven days. The differences may be attributed to modifiers diffusion capacity and the release of particles from cement [65]. The antibiotic could more effectively diffuse (Figure 7), and thus it would kill bacteria in the nearby environment. However, in the case of nanosilver, the effect was more emphasized on the cement surface. The obtained results are in line with the literature [66,67,68], although some reports suggested that nanosilver is ineffective [69,70]. In our studies, the effective surface antibacterial effect of NpAg-BC could be assumed. More detailed analysis regarding the bactericidal effectiveness of nanosilver was carried out in our previous research [26].

#### 3.4.2. Results of Cytocompatibility Studies

**Figure 8:**
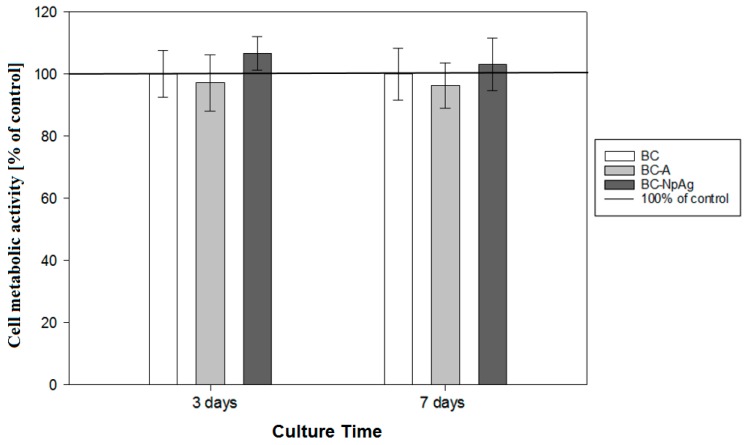
MC3T3-E1 metabolic activity on the surface of BC coatings at day 3 and 7 culture. Results are expressed as % change in cell metabolic activity on modified bone cements compared to the cell metabolic activity on pure bone cement, assumed as 100% (n = 3; mean ± SD; * significantly different from unmodified bone cement—*p* < 0.05).

Figure 8 presents the metabolic activity of MC3T3-E1 cells after three and seven days culture on the surface of bone cement coating. 

**Figure 9:**
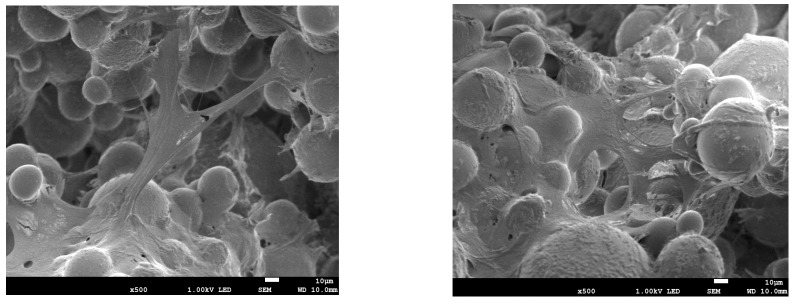
MC3T3-E1 cells adhesion to the surface of bone cement coatings after 7 days (the presented pictures are representative for all types of BCs).

Bone cements coatings containing antibiotic or nanosilver did not affect MC3T3-E1 cells metabolic activity when compared to pure bone cement. Thus, the proposed modifications of BC were assumed cytocompatible. The MC3T3-E1 cells adhered well to both investigated bone cements and example pictures, as shown in Figure 9.

The present results are consistent with our previous study with human dental pulp stem cells and blood cells [26] and the research of others [71,72,73]. Thus, coating spacer with bone cement modified with antibiotic or nanosilver (up to 3%) does not deteriorate the overall biocompatibility of the material construct. 

## 4. Conclusions

In this study, we tried to eliminate one of the main reason of complications after implantation of spacers by applying 3D printing and surface modification of titanium alloys after their processing. The specimens were sandblasted, ground or etched to improve the surface roughness of Ti13Zr13Nb alloys. The titanium alloy was selected based on its excellent properties dedicated for biomedical applications. In order to improve the spacer’s longevity we used selective laser melting methods, which could manufacture the personalized solutions. Moreover, we evaluated the antibiotic- and nanosilver-enriched coatings on the titanium alloy surfaces. Our results indicated that the applied technology of the fabrication of titanium alloy specimens affects their surface parameters such as roughness, wettability, nanohardness, surface energy and Young’s modulus. Also, bone cement adhesion to such titanium alloy surfaces depended on their initial treatment. The best BC adhesion was found for titanium alloy specimens that displayed high nanohardness and roughness. There was no correlation of adhesion strength to the wettability or surface energy of titanium alloys. The antibacterial additives to BC, such as gentamicin and nanosilver allowed for adequate anti-bacterial protection of the obtained spacers while maintaining their biocompatibility. Such modified BC had different effects on their adhesive capacity to the titanium alloy specimens or their own cohesion. The modified cements adhered poorly to the ground titanium alloy specimens, but they adhered better to rough ones. Sandblasted or etched titanium alloy specimens were the best for bone cement adhesion, which was probably due to their high roughness, nanohardness, as well as the removal of SLM production residues. 

Overall, the higher adhesion strength of bone cement coating to the SLM specimens is a good precondition for the SLM application in the production of metal-polymer implants for tissues with heavy loads. Although titanium alloy and bone cements present separately attractive physical and mechanical properties, there is still a need for improving their bonding interface for the longevity of spacers. We believe that this study will prompt further research investigating other titanium alloy surface modifications, such as thermo-chemical treatment or laser processing. The additional mechanical testing, such as shearing force adhesion or fatigue endurance, should also be investigated. Furthermore, the effectiveness of such spacers should be evaluated in body fluids followed by in vivo examination of spacers in animal models.

## Figures and Tables

**Table 1:** Chemical composition of bone cements used for titanium specimens coating.

	Unmodified Bone Cement/BC/	Antibiotic-Loaded Bone Cement/A-BC/	Bone Cement Modified with Nanosilver/NpAg-BC/
	**Powder component [% w/w]**		
Polymethyl methacrylate	84.30	83.05	83.05
Barium sulphate	13.00	12.80	12.80
Benzoyl peroxide	2.70	2.65	2.65
Gentamicin sulphate	X	1.5	X
Nanosilver	X	X	1.5
	**Liquid component [% w/w]**		
Methyl Methacrylate	99.10	99.10	99.10
N, N-dimethyl-p-toluidine	0.90	0.90	0.90
Hydroquinone	75	75	75
Methyl Methacrylate	99.10	99.10	99.10

**Table 2:** Surface roughness parameters: Ra, Rz, Rp of surface-modified titanium specimens measured by profilometer (n = 5; data are the means ± SD; * significantly different from control A (*p* < 0.05); ^#^ significantly different from control B (*p* < 0.05)).

Surface Roughness Parameters (µm)					
	Solid Bar/Control A/	Untreated SLM/Control B/	Sandblasted SLM	Etched SLM	Ground SLM
**Ra**	0.14 ± 0.02 ^#^	10.13 ± 0.20 *	10.79 ± 0.36 *	8.82 ± 0.99 *^,#^	1.38 ± 0.24 *^,#^
**Rz**	1.71 ± 0.28 ^#^	55.95 ± 3.49 *	54.64 ± 0.79 *	45.92 ± 5.09 *^,#^	7.32 ± 6.90 ^#^
**Rp**	1.30 ± 0.20 ^#^	28.78 ± 1.89 *	30.52 ± 0.26 *	26.00 ± 3.39 *^,#^	3.68 ± 0.57 ^#^

**Table 3:** Surface wettability of the investigated surface-modified titanium alloy specimens determined by the measurements of the contact angle of distilled water (n = 5; data are the means ± SD; * significantly different from control A (*p* < 0.05); ^#^ significantly different from control B (*p* < 0.05)).

Surface Wettability–the Value of Contact Angle (°)				
Solid Bar/Control A/	Untreated SLM/Control B/	Sandblasted SLM	Etched SLM	Ground SLM
76.4 ± 7.1 ^#^	120.3 ± 2.3 *	68.3 ± 5.23 ^#^	81.2 ± 6.6 ^#^	50.9 ± 4.8 *^,#^

**Table 4:** The surface energy surface-modified titanium alloy specimens. The results are displayed as mean values of contact angles; * significantly different from control A (*p* < 0.05); ^#^ significantly different from control B (*p* < 0.05).

Surface Energy (mN/m)					
	Solid Bar/Control A/	Untreated SLM/Control B/	Sandblasted SLM	Etched SLM	Ground SLM
**Ɣs**	41.11	48.69	51.39 ^#,^*	38.40 ^#,^*	67.25 ^#,^*
**ƔsD**	33.84	45.07	47.27	34.71	36.94
**ƔsP**	7.27	3.62	4.12	3.69	30.31

**Table 5:** Nanomechanical properties (nanohardness and reduced Young’s modulus) of investigated surface-modified titanium alloy specimens (n = 25; * significantly different from control A (*p* < 0.05); ^#^ significantly different from control B (*p* < 0.05)).

	Solid Bar/Control A/	Untreated SLM/Control B/	Sandblasted SLM	Etched SLM	Ground SLM
**Nanohardness (GPa)**	5.15 ± 1.12 ^#^	3.94 ± 1.76 *	2.43 ± 1.46 *^,#^	0.41 ± 0.19 *^,#^	6.77 ± 2.14 *^,#^
**Reduced Young’s modulus(GPa)**	133.50 ± 17.36 ^#^	62.93 ± 26.30 *	53.15 ± 24.15 *	24.00 ± 9.92 *^,#^	110.39 ± 20.26 *^,#^
**H/Er**	0.038 ± 0.004 ^#^	0.066 ± 0.025 *	0.052 ± 0.038	0.018 ± 0.006 *^,#^	0.060 ± 0.009 *
**H^3^/Er^2^ (GPa)**	0.008 ± 0.004 ^#^	0.020 ± 0.017 *	0.016 ± 0.003	0.0002 ± 0.0015 *^,#^	0.027 ± 0.015 *

**Table 6:** Cohesion of bone cement coating (n = 10; * significantly different from BC—*p* < 0.05; ^#^ significantly different between group—*p* < 0.05).

BC (mN)	A-BC (mN)	NpAg-BC (mN)
81.2 ± 19.4	84.9 ± 13.8 ^#^	122.0 ± 11.5 *^,#^

**Table 7:** Adhesion of bone cements to surface-modified titanium alloy specimens (n = 5; data are the means ± SD; * significantly different from control A (*p* < 0.05); ^#^ significantly different from control B (*p* < 0.05)).

Adhesion of Bone Cements to Surface-Modified Titanium (N)					
	Solid Bar/Control A/	Untreated SLM/Control B/	Sandblasted SLM	Etched SLM	Ground SLM
**BC**	86.8 ± 53.1 ^#^	1432.7 ± 79.5 ^*^	>2000 ^x,#,^*	>2000 ^x,#,^*	827.9 ± 276.6 *^,#^
**A-BC**	168.3 ± 92.6 ^#^	1929.1 ± 18.8 ^*^	>2000 ^x,^*	>2000 ^x,^*	61.6 ± 46.7 ^#^
**NpAg-BC**	10.0 ± 0.5 ^y,#^	1980.3 ± 20.3 ^*^	>2000 ^x,^*	>2000 ^x,^*	69.1 ± 25.6 ^*,#^

^x^ The rupture of adhesive-specimens connection with an average strength about 2000 ± 234.5 N. ^y^ The rupture of cement-specimens connection when the preload was applied.

**Table 8:** Bacterial growth inhibition for untreated SLM titanium alloy specimens coated with bone cements (n = 3; data are the means ± SD; * significantly different from control (*p* < 0.050; ^#^ significantly different between modifications (*p* < 0.05)).

Time (h)	The Bacterial Growth Inhibition Zone (mm)					
	BC		A-BC		NpAg-BC	
	*Stap. aureus*	*E. coli*	*Stap. aureus*	*E. coli*	*Stap. aureus*	*E. coli*
24	0	0	10.2 ± 0.2 *^,#^	9.9 ± 0.6 *^,#^	1.1 ± 0.2 *^,#^	1.0 ± 0.3 *^,#^
72	0	0	10.1 ± 0.5 *^,#^	9.2 ± 0.5 *^,#^	1.1 ± 0.1 *^,#^	1.2 ± 0.3 *^,#^
168	0	0	10.4 ± 0.4 *^,#^	10.5 ± 0.4 *^,#^	1.2 ± 0.4 *^,#^	1.6 ± 0.5 *^,#^
